# Unveiling the potential for an efficient use of nitrogen along the food supply and consumption chain

**DOI:** 10.1016/j.gfs.2020.100368

**Published:** 2020-06

**Authors:** Sara Corrado, Carla Caldeira, Gema Carmona-Garcia, Ina Körner, Adrian Leip, Serenella Sala

**Affiliations:** a European Commission, Joint Research Centre (JRC), Via E. Fermi, 2749, 21027, Ispra, VA, Italy; b Hamburg University of Technology, Institute of Wastewater Management and Water Protection, Bioresource Management Group, Eißendorfer Straße 42 (M), 21073, Hamburg, Germany

**Keywords:** Nitrogen flows, Food waste, Mass balance, Food system, SDG 12, Nitrogen circularity

## Abstract

Ensuring global food security is one of the challenges of our society. Nitrogen availability is key for food production, while contributing to different environmental impacts. This paper aims firstly to assess nitrogen flows and to highlight hotspots of inefficient use of nitrogen along the European food chain, excluding primary production. Secondly, it aims to analyse the potential for reducing the identified inefficiencies and increase nitrogen circularity. A baseline and three scenarios-reflecting waste targets reported in EU legislation and technological improvements- are analysed. Results highlighted a potential to reduce reactive nitrogen emissions up to more than 45%. However, this would imply the conversion of reactive nitrogen in molecular nitrogen, such as urea, before re-entering in the food chain. Techniques to harvest reactive nitrogen directly from urine and wastewater are considered promising to increase nitrogen use efficiency along the food chain.

## Highlights

• Nitrogen flows from post-farm gate to consumption in food system were investigated.
• Only 45% of the nitrogen flows in the post-farm gate food system ends up as ingested N.
• Emissions of reactive nitrogen can be reduced up to 70% in tested scenarios.
• Tertiary wastewater treatment plants reduce reactive nitrogen emissions but not contribute to N circularity.
• Nitrogen circularity can be increased with innovative techniques capturing reactive nitrogen.

## 1 Introduction

Food production is expected to considerably rise in the next years to satisfy the needs of the increasing world population (Foresight, 2011). This is problematic, given that the current food system is responsible for several impacts on the environment, contributing to climate change, water scarcity, eutrophication and biodiversity loss (Sala et al., 2017; Leip et al., 2015; Willett et al., 2019). A decoupling of food supply from environmental impacts is, therefore, an essential sustainability goal. In this context, the biogeochemical flows of nitrogen (N) and phosphorus (P) are of particular relevance as they are among the processes for which planetary boundaries are already substantially transgressed (Steffen et al., 2015). Reducing losses of nutrients from food systems could contribute to decreasing environmental impacts, while keeping the nutrients for consumption or food production. Indeed, the food system is one of the chief cause of the alteration of the N cycle, e.g. due to the creation and emission to the environment of reactive nitrogen (Nr), such as nitrogen oxides, ammonia, nitrous oxide, and nitrates, mainly linked to the production and use of fertilizes (Erisman et al., 2008; Sutton et al., 2011). While Nr is essential for supporting human life and enabling the production of sufficient food, its emission to the environment is responsible for several environmental impacts (Galloway et al., 2008; Leip et al., 2015). For example, nitrates emitted into water contribute to the eutrophication of water bodies. Nitrous oxide emissions account for about 6% of the anthropogenic emissions of greenhouse gases in the European Union (EU) (EEA, 2018). Moreover, ammonia and nitrogen oxides contribute directly to acidification and terrestrial eutrophication and, reacting with other pollutants, to the formation of secondary particulate matter and photochemical ozone.

The situation is aggravated by food waste generation, which is estimated at one third of food being discarded globally (Foley et al., 2011; FAO, 2013). At the same time, a vast potential for improvement exists for reducing the edible fraction of food waste while increasing the cascade use of resources. Food waste reduction is one of the targets of the United Nations Sustainable Development Goal 12 (SDG 12) (UN, 2015). Several international initiatives have been put in place to support the achievement of SDG target 12.3 on food waste reduction. The “Champions 12.3”, for example, is a coalition of governments, business, international organisations, research institutions with the aim of accelerating the progress toward SDG 12.3 target (WRI and Ministry of Economic Affairs of the Netherlands, 2016). In the EU, food waste reduction is also advocated in specific policies targeting the need of moving towards circular economy, i.e. maximising the recycling of materials, such as the Circular Economy Action Plan (EC, 2020), the Bioeconomy Strategy (EC, 2018), and the Waste Framework Directive (European Parliament and Council, 2018)

A number of studies addressing the link between food waste and nutrient flows have been published over time. For example, Grizzetti et al. (2013) and Vanham et al. (2015) assessed the contribution of food waste to N pollution. Notarnicola et al. (2017) studied the impacts of EU food consumption on 16 environmental impact categories, among which those due to N compounds appear to have an important contribution (e.g. eutrophication and acidification). Brancoli et al. (2017) quantified the environmental impacts associated with food waste generation in supermarkets. Estimates of food waste generation are provided e.g. at the international (Gustavsson et al., 2011), European (Caldeira et al., 2019), or national (Beretta et al., 2013) scales, or considering specific business units (e.g. four supermarkets as in Eriksson et al., 2012). N flows within the food system cover the quantification of nitrogen footprints in food product categories (Leip et al., 2014), the distribution of Nr emissions along the food system (Leip et al., 2014; Bodirsky et al., 2014), or the estimation of N flows within national food systems (Antikainen et al., 2005; Le Noë et al., 2017). A main conclusion common to all these studies is that the food system is characterised by huge inefficiencies, both in terms of food waste generation and of N management along the food chain, and that a high potential for improvement exists.

Existing literature has not yet addressed food waste and nitrogen management using a comprehensive mass balance to identify the potential for improving N circularity, i.e. the potential of increasing the cascade use of N that is in food, food waste, and by-products. Therefore, by connecting these two dimensions, this paper aims firstly to identify the main hotspots of inefficient use of N along the food system (excluding primary production), taking the EU in 2011 as case study. Secondly, by testing the efficacy of different scenarios, it aims to contribute to unveil the potential for reducing the identified inefficiencies of the food system, limiting N losses and improving N circularity, where possible. Given the broad geographical scope of the study, results should be interpreted from a strategic perspective for the EU, rather than as a precise estimation of nitrogen flows at local level.

## 2 Materials and methods

**Fig. 1:**
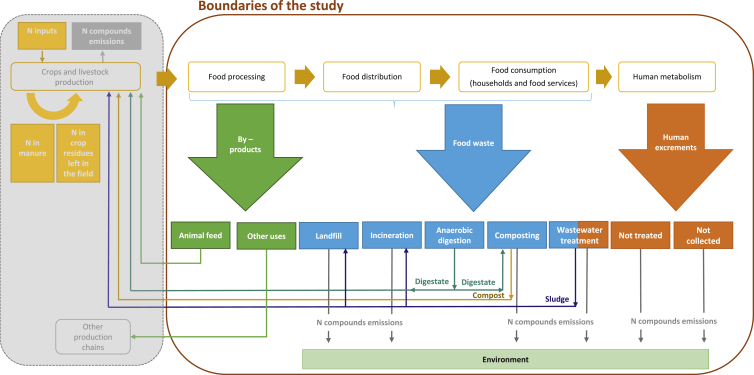
Source and destinations of N flows in the food system, focusing on the flows within the post farm gate boundaries. The figure refers to the Baseline and Improved scenarios.

In this study, we analysed the N flows within the EU food system, as illustrated in Fig. 1. The system boundaries of the study include post-farm gate stages of the food chain, i.e. processing, distribution, and consumption, and human metabolism, intended as the processes of human digestion of food and excretion of residues as well as waste management. N use efficiencies at primary production were not included in this study because they are extensively studied elsewhere (see e.g. Hutchings et al. (this issue)).

The overall quantification of N flows was carried out in four steps that are explained in detail in the following sections (from 2.1.1 to 2.1.4). As a last step, we verified the reliability of the overall mass balance by comparing the resulting protein intake with other data sources for the same information (section 2.1.5).

Since different definitions of “food waste” exist, we adopted the definition from the EU Waste Framework Directive (European Parliament and Council, 2018), according to which “food waste means all food […] that has become waste”. Other fractions of food removed from the food supply and consumption chain that are not falling under the definition of food waste are reported as by-products.

The sum of N in by-products, food waste, and human excrements is hereafter referred to as ‘total N’, corresponding to the overall amount of N leaving the food chain. The term “destination” refers to waste treatments for food waste and human excrements, and to animal feed or other uses (e.g. biorefineries for by-products).

Taking stock of a recent study on food waste quantification done by Caldeira et al. (2019), 2011 was selected as reference year. Data were collected at EU scale. When data for 2011 were not available, we used data from the closest available year. When EU data were not available, those were collected from national sources. The quantification of N flows was done for four scenarios: (1) Baseline, reflecting the situation in the EU in 2011, (2) Improved, (3) Advanced, and (4) Combined. Subsection 2.1 describes in detail how the Baseline scenario was built, whereas subsection 2.2 explains the other scenarios.

### 2.1 Definition of the Baseline scenario

#### 2.1.1 Nitrogen in food, food waste, by-products, and human excrements

Estimation of N in food, food waste, and by-products was performed for ten food product groups: meat, fish, dairy, eggs, cereals, fruit, vegetables, potatoes, sugarbeets, and oilcrops. The amount of N contained in each food product group at each stage of the food chain was calculated by multiplying the quantities of the given product group reaching that stage by the relative N concentrations (kg_N_/kg). Quantities of consumed food, food waste, and by-products at each stage of the EU food chain were taken from Caldeira et al. (2019). Food waste amounts at processing and consumption were divided in liquid and solid fractions. The share of liquid food waste at processing was calculated as fraction of the amount reported by Caldeira et al. (2019) based on the amount of sludge generated by the NACE activity “Manufacture of food products: beverages and tobacco products”, reported in Eurostat (2019a). Liquid food waste at consumption was estimated as a fraction of uncollected food waste disposed through the sink, as explained in section 2.1.2.

N concentrations were calculated on the basis of data reported by Carmona-Garcia et al. (2017), Britz and Witzke (2014), and USDA (2018) (see Supplementary data).

An overview of food waste, by-products, and food quantities, and the specific N concentrations considered for the Baseline scenario are reported in Table 1.

**Table 1:** Amount of food waste and by-products (wet mass) (Caldeira et al., 2019), and N concentration (Carmona-Garcia et al., 2017; Britz and Witzke, 2014; USDA, 2018) per food product group and stage of the food chain.

		Meat	Fish	Dairy	Eggs	Cereals	Fruit	Vegetables	Potatoes	Sugarbeets	Oilcrops
Food waste (Mt wet y^−1^)	Processing	2.9	3.0	1.0	0.8	2.5	6.0	2.6	2.1	0.0	10.0
	Distribution	1.7	0.3	0.4	0.1	1.7	0.8	0.9	0.3	0.4	0.1
	Consumption	9.0	1.2	5.2	1.3	10.1	10.3	14.5	5.9	1.6	1.7
	- of which in households	7.3	0.7	4.5	1.0	8.0	8.8	12.2	4.9	1.3	1.4
	- of which in food services	1.7	0.5	0.7	0.3	2.1	1.6	2.2	1.0	0.3	0.3
By-products (Mt wet y^−1^)	Processing	26.3	1.5	10.0	0.6	25.6	7.3	2.6	0.0	8.5	14.8
N concentration (gN kg^−1^ wet)	Processing	36.7	33.1	5.6	20.9	27.4	2.0	3.3	3.4	9.1	52.2
	Distribution	32.7	33.1	5.5	19.2	10.2	1.2	3.6	5.0	2.2	34.1
	Consumption	32.7	33.1	6.3	20.9	10.2	1.7	3.5	5.0	1.6	3.6

The amount of N in human excrements was calculated as the difference between the N intake and the N absorbed. The N intake was calculated by multiplying the consumed food reported by Caldeira et al. (2019) by the N content of food taken from CAPRI model (Britz and Witzke, 2014). The N absorption by humans was calculated using an average per capita weight of 70 kg, a nitrogen content in the body of 3%, and an average lifespan of 80 years, resulting in 26 g of absorbed N per person per year (gN p^−1^ y^−1^). This gives an average N excretion of 5.9 kgN p^−1^ y^−1^, equal to 99.6% of the N intake. It is evident that this average estimation entails a degree of uncertainty due to extreme variability of diets, specific characteristics of EU citizens, and rough assumptions on the N absorption. However, it is in line with the amount of N in human excrement (range low-high values equal to 0.8–14.9 kgN p^−1^ y^−1^) reported by Rose et al. (2015), who made a literature review on the characterisation of human faeces and urine in different world countries.

#### 2.1.2 Destinations of food waste and by-products

Food waste at the processing and distribution stages was assumed to be entirely collected, whereas at consumption stage a fraction of food waste was uncollected.

The share of collected solid food waste reaching different destinations was obtained from EU statistics. Food waste is not reported as a standalone category in EU waste statistics and it is partly captured in the European Waste Classification for Statistics (EWC-stat) categories (Eurostat, 2019a, 2017). We assumed that 100% of the category “09.1 Animal waste of food preparation and product”, and 25% of the category “10.1 Household and similar waste” is food waste (Lebersorger and Schneider, 2011). Category “09.2 Vegetal waste of food preparation and products” was assumed to contain predominantly gardening waste. Shares of food waste going to different treatment systems were available, by waste category, from Eurostat statistics on waste treatment (Eurostat, 2019b). The following destinations were considered for the solid fraction:

a) anaerobic digestion and composting (defined in European statistics as “Recovery other than energy recovery”),
b) incineration (sum of “Incineration – disposal” and “Incineration – energy recovery”),
c) landfill (“Deposit onto or into land”).

Anaerobic digestion was further subdivided in the following options: a1) anaerobic digestion with direct application of digestate in agriculture, and a2) and anaerobic digestion with digestate composting and compost application in agriculture. The share going to each of the two destinations was defined according to Gilbert and Siebert (2019).

Fraction of uncollected food waste (including both solid and liquid fractions) were calculated from Kranert et al. (2012) data. They estimated the destinations of household food waste (home composting, sewer, and pet food) in Germany based on the evaluation of various scientific studies. The uncollected food waste, i.e. the fraction of food waste not captured by the communal waste collection systems, was 24% for the household sector. Furthermore we assumed 11% uncollected waste for food services. In both cases, we simplified that half of the uncollected food waste fraction was disposed through the sewage system and the remaining half was used for home composting.

For the liquid fraction of food waste, the same destinations assumed for human excrements were used (see Section 2.1.3).

For the destinations of by-products generated at the processing stage, we used the data reported by Caldeira et al. (2019). Animal feed was the only destination for all the by-products, except for the ones generated by meat and eggs processing, which were used for other purposes, such as fertilisers, pet food, and leather production. In these cases, and in those where N was embedded in compost or digestate, N was classified as “valorised” because it re-enters the food chain or other productive chains.

#### 2.1.3 Destinations of human excrements

In the EU, both fractions from human excrements, faeces and urine, are commonly collected together through the sewage system. The wastewater collection and treatment are regulated by the urban wastewater treatment directive (European Council, 1991), which establishes that the wastewater from cities larger than 2000 inhabitants has to be collected. EEA (2019) provided shares for collected and treated wastewater amounts for the EU, considering five geographical areas. The shares of uncollected and collected but untreated wastewater, and the amount of wastewater going to different wastewater treatment technologies (i.e. primary, secondary, and tertiary treatment) were calculated for the EU as a weighted average based on the population of Member States.

The destination of sewage sludge was taken from Eurostat (2019b). Eurostat reported that in 2011 sewage sludge was recycled in agriculture (53%), sent to landfill (24%), and incinerated (23%).

#### 2.1.4 Environmental losses of N

During waste treatment, emissions of N can occur as non-reactive, i.e. molecular N (N_2_), not harmful for the environment, or reactive N (Nr), including nitrates, ammonia, and nitrogen oxides, responsible for various pollution phenomena. The remaining N that is neither emitted as Nr nor as N_2_ remains in the food waste or in the output of the waste treatments and, in some cases, can be valorised as input either to the food chain or to other productive chains.

Average N emission factors from composting were 23.2% of initial N for ammonia and 0.3% for N leaching (median values reported by Körner et al., 2009). The same N emission factors were considered for the case in which anaerobic digestion is followed by composting, and for home composting. No emissions of N happen during anaerobic digestion, where all the N is largely transformed to ammoniacal N and remains in the digestate.

Emissions from incineration are mainly generated by two types of reactions: oxidation of the nitrogen in the waste (fuel–NO_x_), and oxidation of the molecular nitrogen in the combustion air (thermal-NO_x_). Being the focus of the study on N circularity, only the former component is considered in the assessment. Even though the latter component could arguably be valorised and enter a productive process, for example as fertilizer, we use here a stricter ‘circularity’ concept and limit the assessment on N in food waste. Nitrogen oxides (NO_x_) are partly removed from the flue gas thanks to so called DeNOx technologies, such as selective non-catalytic reduction and selective catalytic reduction. A share of 30% emitted as Nr and 70% emitted as N_2_ was calculated as weighted average of the emissions from the two technologies (details in Supplementary data). The residues form the incineration, e.g. ash, containing part of the nitrogen removed from the flue gas were assumed to be disposed in landfills.

N losses from landfills are highly variable and depend on different elements, such as the type of waste and the climatic conditions. Bacterial activity and related N emissions vary during the lifetime of the landfill. The first phase is characterised by aerobic conditions and N emissions assumed for compost were considered. The following phases, instead, are characterised by lack of oxygen. Emissions of N in these stages were modelled according information reported by Cossu et al. (2005) and Brandstätter et al. (2015). Emissions from landfills are 47.4% of available N, thereof 39.2% as N_2_ and 8.2% as Nr, whereas the remaining 52.6% stays in the body of the landfill.

For incinerated or landfilled sewage sludge and ash, the N emission factors abovementioned for incineration and landfill were used.

We used nitrogen removal efficiencies and N emissions from Bonomo (2008) for primary and secondary wastewater treatments, i.e. respectively 10% and 20% emitted as Nr, and data from Mc Carty (2018) for tertiary wastewater treatment technologies, i.e. 30% of N emitted as Nr, and 51% emitted as N_2_. For all the treatment, the share of N not emitted to the environment stays in the wastewater sludge.

#### 2.1.5 Estimation of protein intake

Protein intake was calculated to crosscheck the reliability of the overall N mass balance, as well as to compare it with N losses and analyse the extent of N circularity in the system analysed. Indeed, the described methodological approach uses information from a large range of data sources, i.e. various statistical databases and coefficient from different literature studies performed in different contexts and for different geographic regions, which may lead to inconsistencies in the results.

Protein intake was estimated considering the N content of food products as given in Section 2.1.1.

### 2.2 Definition of scenarios

In addition to the Baseline scenario (as described in the previous sections) we defined scenarios to assess the potential for improving the N efficiency of the EU food system (excluding primary production), varying the share of food waste and by-products going to different destinations at constant human food intake and the efficiencies of waste treatments. The Improved scenario assumed different levels of food waste reduction and improvements in waste and wastewater management. The Advanced and Combined scenarios included the uptake of innovative techniques for N recovery from wastewater (Kumar and Pal, 2015). For the Improved scenario, we assumed that food waste prevention is possible only at the distribution and consumption stages, where edible food waste generation happens. Indeed, food waste prevention actions focus on the edible fractions of the waste, whereas inedible fractions, such as kernels and bones, being unavoidable, can only be valorised. For processing, where most of the food waste is inedible (De Laurentiis et al., 2018), we assumed that the reduction of food waste is realised by diverting part of the food waste to animal feed. The Improved scenario was mainly defined in light of the targets set by the EU Circular Economy Package (EC, 2015), i.e. 50% food waste reduction for retail and consumption, and overall reduction along the food chain to be achieved by 2030 and the Waste Framework Directive (European Parliament and Council, 2018), establishing that 100% of bio-waste is to be collected separately from other waste streams by 2023. For liquid waste, an improvement in the collection rates of wastewater and in the type of treatment was considered, assuming that the collection rate of central EU (best within EU areas) is reached in the entire EU (EEA, 2019). Furthermore, this scenario assumes an increased denitrification efficiency in wastewater tertiary treatment and lower Nr emissions from composting.

The Advanced scenario assumes that 75% of wastewater in the Baseline scenario is treated with innovative N recovery techniques and that the effluent water is sent to tertiary wastewater treatment. The efficiency of the N recovery process was considered to be 77% (Pradhan et al., 2017). The Combined scenario puts together the advantages of the Improved and Advanced scenarios respectively for waste reduction and N recovery.

The assumptions for each scenario are summarised in Table 2, whereas a summary of the share of food waste going to the different destinations in the four scenarios is given in Table 3 and the share of Nr and N_2_ emissions is reported in Table 4.

**Table 2:** Summary of the elements considered in the three analysed scenarios. Improved, Advanced, and Combined scenarios are defined, highlighting differences compared to the Baseline scenario.

		Baseline scenario	Improved scenario	Advanced scenario	Combined scenario
**Processing**	**Produced amount**	Solid waste: Status quo in 2011 based on Caldeira et al. (2019) Liquid waste: based on Eurostat (2019a)	Solid and liquid waste: - 10% food waste generation (sent to animal feed) compared to Baseline scenario	Solid waste: Status quo in 2011 based on Caldeira et al. (2019) Liquid waste: based on Eurostat (2019a)	Solid and liquid waste: - 10% food waste generation (sent to animal feed) compared to Baseline scenario
	**Destination**	Solid waste: Based on Eurostat waste statistics (2019a, 2019b) Liquid waste: see "Human metabolism"	Solid waste: -100% landfill, −80% incineration, waste equally diverted to composting and anaerobic digestion. Liquid waste: see "Human metabolism"	Solid waste: Based on Eurostat (2019a, 2019b). Liquid waste: see "Human metabolism"	Solid waste: -100% landfill, −80% incineration, waste equally diverted to composting and anaerobic digestion Liquid waste: see "Human metabolism"
**Distribution**^a^	**Produced amount**	Status quo in 2011 based on Caldeira et al. (2019)	- 50% food waste generation (prevented)	Status quo in 2011 based on Caldeira et al. (2019)	- 50% food waste generation (prevented)
	**Destination**	Based on Eurostat (2019a, 2019b)	Solid waste: -100% landfill, −80% incineration, waste equally diverted to composting and anaerobic digestion	Based on Eurostat (2019a, 2019b)	Solid waste: -100% landfill, −80% incineration, waste equally diverted to composting and anaerobic digestion
**Consumption**	**Produced amount**	Status quo in 2011 based on Caldeira et al. (2019)	- 50% food waste generation (prevented) Liquid waste: see "Human metabolism"	Status quo in 2011 based on Caldeira et al. (2019)	- 50% food waste generation (prevented) Liquid waste: see "Human metabolism"
	**Destination**	Based on Eurostat (2019a, 2019b)	Solid waste: -100% landfill, −80% incineration, waste equally diverted to composting and anaerobic digestion Liquid waste: see "Human metabolism"	Solid waste: Based on Eurostat (2019a, 2019b) Liquid waste: see "Human metabolism"	Solid waste: -100% landfill, −80% incineration, waste equally diverted to composting and anaerobic digestion Liquid waste: see "Human metabolism"
**Human metabolism**	**Produced amount**	Calculated. Verified with data from Rose et al. (2015)			
	**Destination - wastewater**	Based on EEA (2019)	Based on EEA (2019)	75% to N recovery, effluent sent to tertiary treatment, 25% as in the "Baseline" scenario	75% to N recovery, effluent sent to tertiary treatment, 25% as in the "Improved" scenario
	**Destination - sewage sludge**	Based on Eurostat (2019b)	- 100% landfill, +20% use in agriculture. The remaining quantity sent to incineration	Based on Eurostat (2019b)	- 100% landfill, +20% use in agriculture. The remaining quantity sent to incineration

a Only solid waste was considered at distribution.

**Table 3:** Shares of food waste and human excrements going to different destinations.

	Scenario	Other uses	Wastewater				N recovery (%)	Composting (%)	Anaerobic digestion (%)		Incineration (%)	Landfill (%)
			Collected without treatment and non-collected (%)	Primary treatment (%)	Secondary treatment (%)	Tertiary treatment (%)			Direct application of digestate (%)	Composting of digestate (%)		
**Food waste Processing**	Baseline	–	0.2	0.2	2.4	7.1	–	27.5	8.2	19.2	19.8	15.3
	Improved	–	0.1	0.0	1.9	7.9	–	43.0	12.9	30.1	4.0	0.0
	Advanced	–	0.2	0.1	0.6	1.8	7.3	27.5	8.2	19.2	19.8	15.3
	Combined	–	0.1	0.0	0.5	2.0	7.4	43.0	12.9	30.1	4.0	0.0

**Food waste Distribution**	Baseline	–	–	–	–	–	–	30.5	9.1	21.3	22.0	17.0
	Improved	–	–	–	–	–	–	47.8	14.3	33.5	4.4	0.0
	Advanced	–	–	–	–	–	–	30.5	9.1	21.3	22.0	17.0
	Combined	–	–	–	–	–	–	30.5	9.1	21.3	22.0	17.0

**Food waste Household**	Baseline	11.5	0.3	0.3	2.8	8.2	–	10.8	3.2	7.5	28.6	26.9
	Improved	11.5	0.1	0.0	2.3	9.2	–	35.6	10.7	24.9	5.7	0.0
	Advanced	11.5	0.3	0.1	0.7	2.0	8.4	10.8	3.2	7.5	28.6	26.9
	Combined	11.5	0.1	0.0	0.6	2.3	8.6	35.6	10.7	24.9	5.7	0.0

**Food waste Food services**	Baseline	5.8	0.1	0.1	1.4	4.1	–	27.0	8.1	18.9	19.5	15.1
	Improved	5.8	0.0	0.0	1.1	4.6	–	42.3	12.7	29.6	3.9	0.0
	Advanced	5.8	0.1	0.0	0.3	1.0	4.2	27.0	8.1	18.9	19.5	15.1
	Combined	5.8	0.0	0.0	0.3	1.1	4.3	42.3	12.7	29.6	3.9	0.0

**Human excrements**	Baseline	–	12.8	2.2	21.4	63.6	–	–	–	–	–	–
	Improved	–	4.1	0.1	18.9	76.9	–	–	–	–	–	–
	Advanced	–	12.8	0.5	5.4	15.9	65.4	–	–	–	–	–
	Combined	–	0.0	0.0	4.7	19.2	71.9	–	–	–	–	–

**Table 4:** Share of nitrogen emissions as reactive nitrogen (Nr) and molecular nitrogen (N_2_) in the different scenarios analysed. Percentages are referred to the N entering the system.

	Nr				N_2_				Literature sources on which scenarios are built
	Baseline	Improved	Advanced	Combined	Baseline	Improved	Baseline	Combined	
Wastewater - collected without treatment	100%	100%	100%	100%	0%	0%	0%	0%	Author's Assumption

Wastewater - primary treatment	90%	90%	90%	90%	0%	0%	90%	90%	Bonomo (2008)

Wastewater - secondary treatment	75%	75%	75%	75%	0%	0%	75%	75%	Bonomo (2008)

Wastewater - tertiary treatment	30%	10%	30%	10%	51%	71%	30%	10%	Mc Carty (2018)

N recovery	7%	2%	7%	2%	12%	16%	7%	2%	Pradhan et al. (2017)

Composting	24%	19%	24%	19%	2%	2%	2%	2%	Körner et al. (2009).

Anaerobic digestion + direct application	0%	0%	0%	0%	0%	0%	0%	0%	

Anaerobic digestion + composting	24%	19%	24%	19%	2%	2%	2%	2%	As composting

Incineration	30%	30%	30%	30%	0%	0%	0%	0%	

Landfill	8%	7%	8%	7%	39%	41%	39%	41%	Cossu et al. (2005) and leachate sent to tertiary treatment

Home composting	5%	5%	5%	5%	54%	54%	54%	54%	Andersen et al. (2011)

### 2.3 Sensitivity analysis

The parameters considered in this study, such as the share of collected waste and the emissions from waste treatments, may considerably vary depending on context-specific conditions. In order to take into consideration such variability, a sensitivity analysis was performed, assuming a 20% variation of aspects related to share of collected food waste and wastewater, efficiency of wastewater treatments, emissions from wastewater treatments, and N recovery. For more details see the supplementary data.

## 3 Results

This section is structured as follows: firstly the hotspots of N loss are highlighted with reference to the Baseline scenario (section 3.1), afterwards the analysed scenarios are put in perspective with the Baseline (section 3.2) and with N ingested by humans (section 3.3) to highlight the potential for improving N circularity and reducing Nr emissions.

### 3.1 Hotspots of nitrogen loss along the EU food system (excluding primary production)

**Fig. 2:**
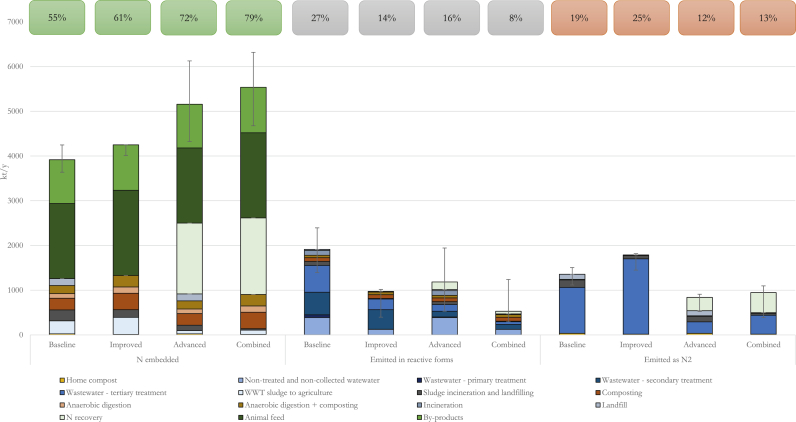
Fate of total food supply N embedded in the outputs of different destinations, and emitted as Nr and N_2_ for the different scenarios. Percentages in the boxes give the average share of N embedded, Nr and N_2_ on the total N. Error bars correspond to the average minimum and maximum results from the sensitivity analysis.

Fig. 2 reports the overall results for the different scenarios disaggregated into embedded N, Nr, and N_2_. Detailed results are reported in Supplementary data. In the Baseline scenario, total N was equal to 7187 kt N y^−1^. 3917 kt N y^−1^ were embedded in by-products, outputs of waste treatment, and landfills, 1913 kt N y^−1^ were emitted as Nr, and 1356 kt N y^−1^ were emitted as N_2_. Fig. 2 shows that in the Baseline scenario about 45% of the total N is valorised mainly as feed or other bio-based products. These two flows of N are coming from the recycling of food by-products generated at processing.

Wastewater treatments, instead, were identified as a hotspot for Nr emissions, being responsible for 61% (1167 kt N y^−1^) of the total Nr emissions. Despite the higher N removal efficiency, tertiary treatment was the main contributor to Nr emissions (600 kt N y^−1^) due to its larger diffusion. It was followed by secondary treatment (505 kt N y^−1^) and untreated or uncollected wastewater (384 kt N y^−1^). Emissions of N_2_ (1356 kt N y^−1^) are almost entirely coming from tertiary treatments (1027 kt N y^−1^).

The sensitivity analysis highlighted that, in relative terms, the variability of parameters influenced more the emissions of Nr rather than the N embedded and higher variations are associated to the Advanced and Combined scenarios. More details on the results of the sensitivity analysis are reported in the Supplementary data.

### 3.2 Potential for increasing nitrogen circularity

The scenario analysis highlighted that N recovery can be increased up to more than 40% (1621 kt N y^−1^), and Nr emissions can be reduced up to 70% (1383 kt N y^−1^), compared to the Baseline. As expected, better results were obtained for the Combined scenario.

The Improved scenario, mainly focused on food waste reduction and partly on the technological improvement of waste and wastewater treatment, mainly reported a benefit in terms of reduction of Nr emissions (−49%; −938 kt N y^−1^), but only a slight improvement in nitrogen valorised (+9%) and a considerable increase in N_2_ emissions (+32%), due to the increased share of tertiary wastewater treatment that converts nitrates to N_2_. Conversely, the Advanced scenario showed a considerable improvement in terms of N recovered (+32%), reduction of N_2_ emissions (−38%) with a lower reduction of Nr emissions (−38%). The combination of the two, resulted in a win-win situation, optimising Nr valorisation (+41%), Nr and N_2_ emissions (respectively −72% and −30%).

The main hotspots for Nr emissions in the Improved scenario was secondary wastewater treatment (16% of total N), followed by wastewater tertiary treatment (9% of total N). In the Advanced scenario, not collected and untreated wastewater was the main contribution (19% of total N), followed by N recovery (8% of total N). In the Combined scenario the shares of Nr emissions from untreated and uncollected wastewater (8%), secondary wastewater treatment (7%), and composting (6%) were quite similar.

### 3.3 Protein intake and overall nitrogen flows

Per capita protein intake is estimated to be 101 g protein p^−1^ day^−1^ (details in Supplementary data).

**Fig. 3:**
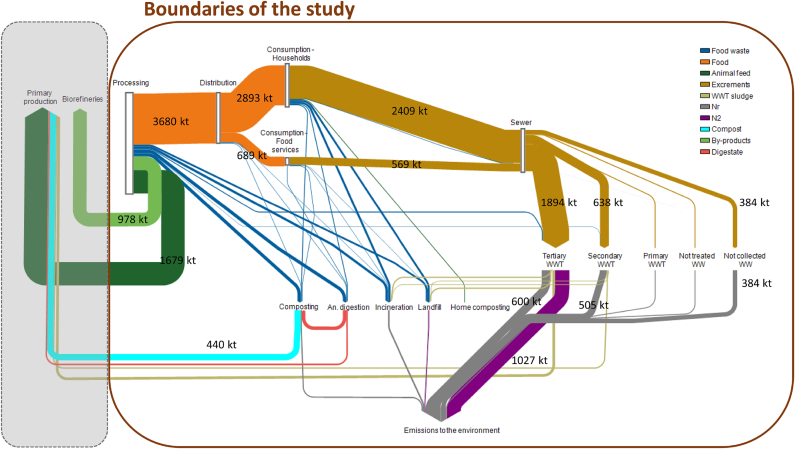
Sankey diagram of the N flows along the EU food system (kt N y^−1^). Flows lower than 20 kt N y^−1^ are not displayed in the figure. Quantities are reported explicitly for flows bigger than 300 kt N y^−1^.

The efficiency of the EU food system in delivering N for human consumption is low, i.e. the ratio between N intake and total N ranges from 41% to 42% for the different tested scenarios. The amount of N ingested by humans is thus lower than the amount of N in food waste and by-products, with the ratio ranging between 0.71 in the Baseline and 0.74 in the Improved scenario. However, N ingested is higher than the losses of N to the environment, with the ratio ranging from 0.9 (Baseline) to 2.0 (Combined) for total (Nr and N_2_) emissions, and from 1.6 (Baseline) to 5.6 (Combined), when referring only to Nr emissions. An overview of all the N flows of the Baseline scenario is reported in the Sankey diagram in Fig. 3.

## 4 Discussion and outlook

This section includes a summary of the key messages from the study and a comparison with similar studies.

### 4.1 Key messages from the study

The analysis of the N flows in the EU food system (excluding primary production) highlighted that about half of the total N is valorised, either as by-product, compost, or digestate. Particularly, the processing stage is a hotspot in terms of food waste and by-products generation. However, the processing stage presents a relatively high N valorisation rate, thanks to the homogeneity of discarded food fraction which makes its recycling easier. Wastewater treatments are the main hotspots in terms of emissions of Nr compounds, hence it is reasonable to prioritise improvement actions focused on them.

The analysis of scenarios shows a significant potential of improving food waste valorisation and/or reducing N emissions, though some interventions will face economic and/or technological barriers which need to be further investigated. For example interventions foreseen by the current EU legislation on waste and improvement in wastewater treatments (Improved scenario) may reduce Nr emissions up to almost 50% compared to the Baseline, while increasing N_2_ emissions by more than 30%. This is mainly due to the diffusion of tertiary wastewater treatment that allows converting a considerable fraction of Nr in N_2_. This has a positive effect on reducing environmental pressures, however, tertiary treatment does not allow closing the N circle and contributes to a linear economic system rather than a circular one (Esculier et al., 2019). The N lost as N_2_ will need to be ‘re-filled’ with new nitrogen through biological nitrogen fixation (e.g. legumes as cover crops), or industrial N fixation. Industrial N fixation is an energy intensive process and creates further emissions (Erisman et al., 2008). However, even if N is retained in sewage sludge, its recycling in agriculture may be subject to some constraints due to the presence of contaminants in sewage sludge that limit its application on agricultural fields (Lamastra et al., 2018).

Alternative techniques to recover N from wastewater treatment (Advanced and Combined scenarios) demonstrated to have a higher potential for increasing N circularity. Nevertheless, technological and economic barriers may limit the implementation of these techniques at the operational scale and need to be further explored. It is worth mentioning that the contribution of untreated and uncollected wastewater is among the top five contributors of Nr emissions in all the scenarios analysed. This means that improving wastewater collection and share of treated wastewater would have a beneficial effect on Nr emissions. However, these kinds of interventions may require a considerable infrastructure investment and further investigations on the cost-benefits and possible trade-offs are needed.

The reduction of food waste as well as the increase of valorised food waste, e.g. through composting, would have positive effects on the decrease of Nr emissions from food waste. One option for improvement is the redirection of food waste already at the point of collection. Currently a large amount is collected with the residual waste, including regions where separate bio-waste collection is in place. Walk et al. (2019) investigated advanced collection systems showing that it is possible to separate food waste at the household level in the kitchen, if the citizens are provided with specific collection vessels. This practice has the potential to redirect a large amounts of food waste away from incineration or landfilling.

Interventions aimed to reduce losses of N in edible food, e.g. through decrease in food waste, should, in principle, be preferred to the ones fostering the valorisation of N in waste, wastewater or by-products. In our study we focus on N flows and the environmental pressure from Nr emissions. However, there might be trade-offs with other environmental impacts. Coupling the analysis of inefficiencies of N use along the food supply and consumption chain with Life Cycle Assessment (LCA) would be an effective approach to identify such possible trade-offs. Indeed, LCA calculates the environmental impacts generated by a product or service along its life cycle, including elements, such as emissions due to energy production and application of compost on the field, that are not considered in this study.

This study is characterised by a broad range of elements of variability. Food waste generation, N content of food, and N emissions are highly context-specific and may considerably vary also within the same country. Having a broad geographical scope, this study does not capture these context-specific differences. Hence, the results of this study should be considered as an overall picture of the inefficiencies of the EU food system related to N management.

### 4.2 Comparison with other studies

Overall, the results of this study are in line with the ones of previous studies. Leip et al. (2014) found that in the EU in 2004, 2.3 Mt of N were emitted to the sewage system due to human metabolism, corresponding to the amount estimated in this study, despite a different reference year. In addition, the authors calculated that 2.8 Mt of N were contained in food waste and by-products generated at processing, which is smaller than our result of 3.5 Mt. This could be due to the different quantities of food waste and by-products and to different N content considered for food waste and by-products. Leip et al. (2014) based their study on the results of the CAPRI and MITTERRA models, whereas our study is based on Caldeira et al. (2019), who considered other statistical and literature sources for the estimation on food waste. In addition, in our study different N contents were considered for food waste, by-products, and food products, whereas Leip et al. (2014) considered a 25% increase for food waste compared to food products, as conservative approach.

Our estimate for N in food waste at consumption in households (484 kt N y^−1^) was about 20% higher than the one obtained by Grizzetti et al. (2013) for the EU in 2007 at 400 kt N. The difference between the results could be associated to the adoption of different definitions of food waste and different assumptions: Grizzetti et al. (2013) considered as food waste the discarded edible fraction of food products, no matter what its destination is, whereas in our study we considered as food waste the discarded edible and non-edible fractions of food going to waste treatments.

Finally, the protein intake obtained, equal to 101 g p^−1^ d^−1^ is about 19% higher than the 85 g p^−1^ d^−1^ average protein intake reported by Westhoek et al. (2011) for the EU, but falls within the ranges identified by national nutritional surveys in EU, reporting a range from 67 to 114 g p^−1^ d^−1^ for men and 59–102 g p ^−1^ d^−1^ for women (European Food Safety Authority EFSA, 2012).

## 5 Conclusions

This study analysed the destinations and the fate of N embedded in the EU food system (excluding primary production) in the EU in 2011. The results showed that such food chain generates about the same amount of N in by-products than in the food consumed by humans, plus about half that amount in non-valorised food waste. As reflected in the Improved scenario, the objective of SDG 12.3 to reduce food waste by 50% by 2030 is likely to increase by 9% the amount of N embedded in valorised products and decrease by almost 50% Nr emissions.

Wastewater treatments are the most relevant destination in terms of Nr emissions. The analysis of scenarios showed a considerable potential for reducing emissions of Nr by more than 45% when fully implementing current EU legislation on waste and improving wastewater treatment. This improvement would require: 1) an increase of the share of tertiary wastewater treatment, 2) a reduction of food waste generation, and 3) a decrease of the quantity of incinerated and landfilled food waste. Decreased emissions of Nr would therefore mainly be achieved by converting it to N_2_ or through the plantation of N-fixing cover crops. However, the first option would imply the emissions of N_2_ that will need to be ‘refilled’, for example through the energy-intensive Haber-Bosch process which is associated with Nr and GHG emissions.

A specific action with potential for improving the cascade use of N lies in the application of innovative wastewater treatment systems, where the N is retained and valorised as a quality fertilizer product. Nevertheless, there are aspects not captured by the scenarios analysed in this study that have the potential to further increase the efficiency of N use along the food supply and consumption chain, such as dietary shifts. Hence, the potential for improving the efficiency of N use along the food chain can go beyond the results of this study. Trade-offs between other environmental impacts and benefits should be taken into account when evaluating optimal solutions for improving N use efficiency. Coupling the analysis of inefficiencies in N use along the food chain with LCA could help to identify such trade-offs. In addition, there is the need to further explore possible technological and economic barriers that may hamper the realisation of interventions aimed to increase N circularity.

## Declaration of competing interest

None.
