# Supplementary material for: Unveiling the potential for an efficient use of nitrogen along the food supply and consumption chain
Source: Glob Food Sec. 2020 Jun;25:100368. doi: 10.1016/j.gfs.2020.100368 (PMC7299078; doi:10.1016/j.gfs.2020.100368)
Supplement: Multimedia component 2 [file mmc2.pdf]

Supplementary material 2

Unveiling the potential for an efficient use of nitrogen along the food supply and consumption chain

Contents

1 Nitrogen of food products and food waste 2

2 Nitrogen in excrements 4

3 Nitrogen compounds emissions from incineration 5

4 Sensitivity analysis 7

References 8

1 Nitrogen of food products and food waste

The estimation of N content of food products in primary production stage is based on protein content and on protein-to-N conversion factors given in USDA food composition database (https://ndb.nal.usda.gov/ndb/). Protein content is taken from several sources:

- For cereals, vegetables, potatoes, oil crops and milk: CAPRI model data (Britz and Witzke, 2014; Carmona-Garcia et al., 2017).
- Fruits: USDA food composition database (https://ndb.nal.usda.gov/ndb/). Fruit peel composition is taken from Romelle et al. (2016) (https://www.ehow.com/info_10033568_components-banana-peel.html, http://www.jocpr.com/articles/physicohemical-constituents-of-pineapple-pulp-and-waste.pdf, https://onlinelibrary.wiley.com/doi/pdf/10.1002/jsfa.2740360817). CAPRI fruit crops are citrus, apple, table grapes and other fruits. Data for peels corresponds to orange for 'citrus', apple for 'apple' and average of banana, pawpaw, pineapple and mango for 'other fruits'. We consider that grapes are not peeled, therefore their N content does not vary along the food chain.
- Sugarbeet, sugar and molasse: CAPRI data plus feedipedia (https://www.feedipedia.org/)
- For fish: average of fish and shellfish species from FAO/INFOODS Global food composition database – version 1.0 (uFiSh1.0) – 2016
- For eggs: national food databases of Denmark (https://frida.fooddata.dk/?lang=en), Italy (http://www.bda-ieo.it/wordpress/?page_id=14) and the Netherlands (https://nevo-online.rivm.nl/) plus FAO data (http://www.fao.org/docrep/005/Y4628E/y4628e00.htm#Contents)
- For meat, CAPRI model data (Britz and Witzke, 2014; Carmona-Garcia et al., 2017).

For the calculation of N content in food products and food waste in the following stages, some assumptions are taken on the fractions of the different product types which are discarded. These assumptions are detailed in Table 1.

Table 1: Assumptions in the composition of waste: which are the wasted parts of the products in each stage of the food chain.

	PRODUCTION	PROCESSING	DISTRIBUTION	CONSUMPTION
**Meat**	Non-consumptive parts from slaughterhouses[1]	Non-consumptive parts of rendering processes[2]	Slaughterhouse products used for human consumption[3]	
**Fish**	We assume same N content in all stages			
**Dairy**	Produced cow and sheep milk	Milk derivatives used for feed and in industrial processes [4]	Losses of milk derivatives in the market[5]	Milk derivatives used for human consumption[6]
**Eggs**	Whole egg	Shell	26% egg without shell + 74% whole egg [7]	Shell
Cereals	Raw cereal	Bran	Cereal after processing[8]	
Fruits	Whole fruit	Peels[9]	80% whole fruit and 20% juice (without peel)	Peels
Vegetables	We assume same N content in all stages			
Potatoes	We assume same N content in all stages			
Sugarbeet	Sugarbeet	Molasse	Processed sugar	Processed sugar
Oilcrops	Olives, sunflower, rapeseed, soya and other oil crops	Sunflower, rapeseed and soya cakes	Olive, sunflower, rapeseed and soya oils	Olive, sunflower, rapeseed and soya oils

[1] Non consumed for food or for any other uses. It is mainly manure. We consider pet food as part of the food, as they are products bought and consumed in households.
[2] Category 1 and 2 rendered fat and protein meal (not usable for human consumption due to sanitary reasons). It does not include other rendering products used for other non-food purposes.
[3] Slaughterhouse and rendering products used for human consumption (as fresh or used in the food industry): meat, organs and fat, pet food and other fresh or rendered products used in food industry, such as gelatine, bone meal, meat meal, feather meal, bone meal, blood meal, rendered fat.
[4] Fresh milk products, whole milk powder, skimmed milk powder, cheese, butter, casein, whey powder and concentrated milk.
[5] Fresh milk products, whole milk powder, skimmed milk powder, cheese, butter and cream.
[6] Cow and sheep milk, fresh milk products, whole milk powder, skimmed milk powder, cheese, butter, cream, casein, whey powder and concentrated milk.
[7] According to literature (https://www.eggindustrycenter.org/media/cms/2014_1_VanHorne_EUEconomicsPerspect_D576964DB61F8.pdf), egg processing sector accounts for 26% of egg market (EU-28, 2014). We assume that 26% of eggs are without shell and the remaining 74% of the egg in the market is the entire egg.
[8] Taken from CAPRI cereal module; it is composed partly of white flour, partly wholemeal flour and some addition of bran in cereal-based products. The share of refined flour depends on the specific cereal.
[9] We assume that waste in the processing sector corresponds to peels of fruits that will be transformed into juice. In the distribution sector, we assume that 80% of the fruit in the market is fresh food and 20% is in the form of juice (https://research.rabobank.com/far/en/sectors/regional-food-agri/world_fruit_map_2018.html), while waste in the households corresponds to peels removed from fruits. N composition takes into account N content of peels and peel weights as compared to the whole fruit.

2 Nitrogen in excrements

A part of the consumed food and drink is transformed into human excrements (feces + urine). Regarding the nitrogen from food, only a small part remains in the body, all other is excreted. The amount of nitrogen excreted after human metabolism was subdivided into fractions contained in urine and in feces and calculated based on data from Rose et al. (2015). Rose et al. (2015) investigated human excrements from various geographical locations and provided key parameters for the design of wastewater facilities (Rose et al. 2015; page 1862). Table 2 provides the key design data based on Rose et al. (2015) regarding excrement masses, Table 3 regarding nitrogen. Both contain additionally own calculations into the units relevant for the scenario development.

It has to be mentioned, that the excrement masses and their N contents range widely. For instance Rose et al. (2015; page 1833) reports a range for wet fecal wet matter from 51-796 g per capita per year (g/p/y), and for fecal dry matter from 12- 81 g/p/y. Reported mean values for urine range from 0.6 to 2.6 l/p/d (Rose et al. 2015, page 1850). A total N excretion via urine was reported with means between 2 to 35 g/p/d (Beler-Baykal et al., 2011; cited in Rose et al. page 1852). A variable protein intake was reported to be the predominant reason for the variation in concentrations with the minimum in cases of absence of proteins in food (Rose et al. 2015, page 1852). The urine to feces ratio is 10.9 for wet and 2.0 for dry substance amounts.

Table 2: Mass data for human excrements based on Rose et al., 2015 (pages 1862), their water contents and their overall amounts for EU28

**Excrement part**	**Mass wet**			**Mass dry**			**Water content**
	g/p/d	kg/p/y [C]	**Mt/y [A]**	g/p/d	kg/p/y [C]	**Mt/y [A]**	% FM [E]
Urine	*1400* [B]	511	257	*59*	22	11	96
Feces	*128* [D]	47	23	*29* [D]	11	5	77
Excrements	1528	558	**281**	88	32	**16**	94

A – calculated with the population of EU28 in 2011 (502964837)

B – key design criteria, median (Rose et al. 2015); converted from l/p/d assuming a density of 1 kg/l

C – units converted considering 365 days (d) in a year (y)

D – key design criteria, median (Rose et al. 2015)

E – calculated via wet and dry masses

Table 3. Nitrogen (N) data for human excrements based on Rose et al. (2015) (mass in g/cap &d: page 1862) and their overall N amounts for EU28

**Excrement type**	**N content**	**N mass**				
	% mass dry [E]	gN/ p/d	kgN/ p/y	**ktN/ y**	gN/ kg dry mass [A]	gN/ kg wet mass [B]
Urine	18.6	*11.*	4.0	2019.4	186.4	7.9
Feces	6.2	*1.8*	0.7	330.4	62.1	14.1
Excrements	14.5	*12.8*	4. 7	**2349.9**	248.5	21.9

A – calculated from N mass and dry mass (Table 2)

B – calculated from N mass and wet mass (Table 2)

In feces, the predominant nitrogen compound is protein. Rose at al. (2015; page l862) provided a protein value of 6.3 g/cap & d as design parameter (conversion factor from N into protein: 6.25). Further N containing compounds in feces include ammonia and nitrite (Rose at al., 2015; page 1841). In urine, of the nitrogenous fractions urea is the predominant, making up between 75% and 90% (Lentner, 1981; cited in Rose et al., 2015, page 1852). Creatinine is a further significant nitrogenous fraction in urine, whereas nitrate concentrations are low (Rose et al., 2015, page 1852).

3 Nitrogen compounds emissions from incineration

N compounds emissions depend on the type of DeNOx technology used in the incineration plant. The two most diffused technologies in the EU are selective non-catalytic reduction (SNCR) and selective catalytic reduction (SCR). The emission factors for nitrogen compounds emissions were determined by considering a share of 25% for SNCR, 43% for SCR high-dust, and 32% for SCR low-dust as reported by Doka (2017). The conversion efficiency of NOx for these two technologies were respectively 60% for the SNCR and 85% for SCR (both high-dust and dust).

4 Results

Table 4 reports an overview of the N embedded, Nr emissions and N2 emissions in the different scenarios analysed.

Table 4. Overview of the results of the study in the Baseline (B), Improve (I), Advanced (A), Combined (C) scenarios. Results are reported in kt per year

	N embedded				Nr				N_2			
**Scenarios**	**B**	**I**	**A**	**C**	**B**	**I**	**A**	**C**	**B**	**I**	**A**	**C**
**Home compost**	26	13	26	13	3	2	3	2	35	18	35	18
**Non-treated and non-collected wastewater**	0	0	0	0	384	123	384	123	0	0	0	0
**Wastewater - primary treatment**	0	0	0	0	62	3	15	1	0	0	0	0
**Wastewater - secondary treatment**	0	0	0	0	505	438	126	110	0	0	0	0
**Wastewater - tertiary treatment**	0	0	0	0	600	238	150	59	1027	1685	257	421
**Wastewater treatment sludge to agriculture**	289	380	72	95								
**Sludge incineration and landfilling**	245	170	119	31	89	12	66	14	167	72	123	44
**Composting**	259	368	259	368	82	86	82	86	7	9	7	9
**Anaerobic digestion**	104	139	104	139	0	0	0	0	0	0	0	0
**Anaerobic digestion + composting**	181	258	181	258	57	60	57	60	5	6	5	6
**Incineration**	0	0	0	0	107	15	107	15	0	0	0	0
**Landfill**	155	0	155	0	24	0	24	0	115	0	115	0
**N recovery**	0	0	1584	1711	0	0	170	62	0	0	299	448
**Animal feed**	1679	1905	1679	1905	0	0	0	0	0	0	0	0
**Other products**	978	1017	978	1017	0	0	0	0	0	0	0	0
**Total**	**3917**	**4252**	**5158**	**5538**	**1913**	**975**	**1184**	**530**	**1356**	**1789**	**840**	**946**

5 Sensitivity analysis

Some of the data used as input data to the calculations are dependent on context-specific conditions. In order to take into consideration the effects of such variability, a sensitivity analysis was performed by assuming a 20% variation compared to the average values of the following parameters: the share of collected food waste and wastewater, the efficiency of wastewater treatments, the emissions from wastewater treatments, and N recovery. Table 5 reports an overview of the input data considered in the sensitivity analysis. Table 6 reports an overview of the results of the sensitivity analysis in terms of variation of N embedded and N emitted compared to average results and based on values obtained from input parameters changes, the minimum corresponding to -20% of the average input values and maximum to +20% of the average input values.

Table 5. Overview of the variations in input data considered for the sensitivity analysis

			Baseline/Advanced scenario			Improved/Combined scenario		
		Var.	Average	Min	Max	Average	Min	Max
Share of collected FW and WW	Share of collected food waste @households	20%	76%	71%	100%	76%	71%	100%
	Share of collected food waste @food services	20%	89%	71%	100%	89%	71%	100%
	Share of collected WW	20%	89%	71%	100%	97%	77%	100%
Efficiency of WWT	Share of Nr from primary treatments	20%	90%	100%	72%	90%	100%	72%
	Share of Nr from secondary treatments	20%	75%	90%	60%	75%	90%	60%
	Share of Nr from primary treatments	20%	30%	36%	24%	10%	12%	8%
Emissions from waste treatments	Share of Nr emissions from composting	20%	24%	28%	19%	19%	22%	19%
	Share of Nr emissions from anaerobic digestion	20%	0%	0%	0%	0%	0%	0%
	Share of Nr emissions from an.digestion + composting	20%	24%	28%	19%	19%	22%	15%
	Share of Nr emissions from incineration	20%	30%	37%	24%	30%	37%	24%
	Share of Nr emissions from landfill	20%	8%	10%	7%	7%	8%	5%
N recovery (only for the advanced scenario)	Share of WW send to N recovery	20%	n.a.			75%	60%	90%
	Efficiency of N recovery	20%	n.a.			77%	62%	92%

Table 6. Results of the sensitivity analysis. % indicate the variations compared to the average associated with the minimum and the maximum variation in input data

	Variation MIN

	Baseline	Improved+	Advanced	Combined
N embedded	-7%	-6%	-16%	-16%
Nr emitted	27%	59%	64%	134%
N_2 emitted	-18%	-19%	8%	16%
	**Variation MAX**			
	**Baseline**	**Improved+**	**Advanced**	**Combined**
N embedded	8%	0%	19%	14%
Nr emitted	-25%	-4%	-55%	-44%
N_2 emitted	11%	2%	-37%	-59%

References

Brandstätter, C., Laner, D., & Fellner, J. (2015). Carbon pools and flows during lab-scale degradation of old landfilled waste under different oxygen and water regimes. Waste Manag, 40, 100-111.

Britz, W. and Witzke, H.P. (2014). CAPRI model documentation 2014. Institute for Food and Resource Economics, University of Bonn, Bonn, Germany.

Carmona-Garcia, G., Leip, A., Weiss, F., Witzke, H.P., Verma, M., Philippidis, G. Kuiper, M., 2017. Enhanced modelling of sustainable food and nutrition security: the agri-food commodity and nutrient flows and the food supply chains. SUSFANS project. D9.4, European Commission.

Cossu, R., Raga, R., & Vettorazzi, G. (2005). Carbon and nitrogen mass balance in some landfill models for sustainability assessment. In "Proceedings Sardinia 2005".Tenth International Waste Management and Landfill Symposium, S. Margherita di Pula, Cagliari, Italy, 3-7 October.

Doka, 2017. Location- and waste-specific life cycle inventories of waste treatment.. Excel tool available at https://www.doka.ch/publications.htm (Accessed December 2019).

Romelle, F. D., Ashwini, R. P., & Manohar, R. S. (2016). Chemical composition of some selected fruit peels. European Journal of Food Science and Technology, 4(4), 12-21.

Rose, C., Parker, A., Jefferson, B., Cartmell, E. (2015). The characterization of feces and urine: a review of the literature to inform advanced treatment technology. Critical reviews in environmental science and technology, 45(17), 1827-1879.
